# Shape‐Customizable 3D Corn Husk‐Derived Carbon Evaporator for High‐Performance Solar Desalination

**DOI:** 10.1002/gch2.202600002

**Published:** 2026-05-07

**Authors:** Xidong Suo, Yufan Yan, Jiayu Mu, Kaiyan Hao, Xintong Yu, Hongtao Qiao, Jie Yang

**Affiliations:** ^1^ Department of Chemistry Xinzhou Normal University Xinzhou Shanxi China

**Keywords:** 3D evaporator, corn shuck, enthalpy of vaporization, self‐cleaning, solar desalination

## Abstract

Currently, 3D interfacial evaporators have attracted significant attention due to their superior evaporation performance. However, the shape of carbon‐based 3D evaporators is often constrained by the original form of biomass materials, which limits their practical applications. Herein, we report a novel strategy for fabricating 3D solar‐driven interfacial evaporators with arbitrary shapes (hemisphere, cone, flake, and Z‐type) by integrating carbon powder derived from corn shuck (CS) with binders. The silver‐doped corn‐based carbon (Ag‐CCS) material exhibits exceptional photothermal conversion efficiency, achieving surface temperatures of 153.3°C (dry) and 95.7°C (wet) under 1 sun illumination. Among the 3D evaporators, the Z‐type design demonstrates the highest evaporation rate of 4.42 kg·m^−2^ h^−1^, attributed to its porous structure, hydrophilicity, low evaporation enthalpy of adsorbed water (1286.13 J g^−1^), and efficient ambient energy absorption and thermal management. Outdoor experiments further validate the Z‐type evaporator's superior performance, with a maximum daily water production of 25.1 kg m^−2^ and automatic salt‐cleaning capability over 20 days. This work paves the way for the scalable fabrication of 3D carbon‐based evaporators, offering a viable solution for seawater desalination.

## Graphical abstract

We present arbitrarily shaped 3D interfacial evaporators using silver‐doped corn shuck carbon (Ag‐CCS). The Z‐type design shows the highest evaporation rate (4.42 kg·m^−2^ h^−1^) with excellent outdoor performance, offering a scalable solution for seawater desalination

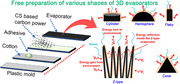

## 1 Introduction

Driven by population growth, industrialization, and environmental pollution, water and energy scarcity have become one of the pressing global challenges [1, 2, 3, 4]. Recently, solar‐driven interfacial desalination has garnered significant interest as a means to mitigate the global growing freshwater and energy shortages [5, 6, 7, 8, 9, 10]. However, there are several pertinent challenges and obstacles to practical implementation and scalability, such as high cost, inadequate solar energy transfer efficiency, and the salt deposits on the photothermal materials [11, 12]. Several strategies have been proposed to address the aforementioned issues [8, 13, 14, 15, 16]. At present, relevant strategies involves as follows: (1) developing photothermal materials from biomass for reducing the cost, including discard corn biomass [17, 18], wood [19, 20], and other agricultural, forestry and daily life by‐products [8, 21, 22, 23]; (2) designing 3D evaporator for improving water evaporation speed and energy efficiency, such as 3D wood based evaporators [19, 20], 3D fabric based evaporators [24, 25], and others 3D structure evaporators [26, 27]; (3) designing new materials and structures to resist salt pollution, for instance, super‐hydrophilic and superhydrophobic materials [22, 28], reverse distillation system [16], cone shape evaporator [12], umbrella shape evaporator [29].

Inspired by the natural plants and trees, many previous studies have proven that designing the evaporator in a three‐dimensional 3D form is an efficient way to enhance the evaporation rate [5, 12, 30, 31, 32, 33, 34, 35]. A 23% enhancement of evaporation rate was achieved to 5.9 kg m^−2^ h^−1^ by a dynamic steering 3D evaporator which composited of graphene wrapped Fe_3_O_4_ nanoparticles [31]. The evaporation rate of a conical 3D wooden based evaporator reached up to 1.9 kg m^−2^ h^−1^ and which is 1.6 times higher than that of 2D evaporator [33]. An arch‐shaped evaporator exhibited an evaporation rate of 2.82 kg∙m^−2^ h^−1^ and good salt collection performance from the bottom surface [36]. Xinchang Pang et al. developed a series of 3D evaporators using CuS as a photothermal conversion material [37, 38]. For example, inspired by the core sheath structure of the tree trunk, a vertical lamellar sodium alginate/aramid fiber supported hydrogel evaporator with CuS coated surface was developed with the outdoor evaporation rate of 12.60 kg m^−^^2^ h^−^^1^, salt resistance, antibacterial properties owing to its cylindrical [38]. However, despite these efforts, the effective utilization of solar energy, high cost and complex preparation process still presents significant challenges to large scale application of solar driven interfacial evaporation. What's more, most carbon‐based 3D evaporators’ shapes and structures were limited by its original shape and structure, which will pose significant impediments to the advancement and application of carbon‐based evaporators.

Herein, various 3D evaporators, namely hemisphere, cone, flaky, and Z‐type, were fabricated via a novel method using glue to bond Ag‐CCS based carbon powder onto the surface of cotton cloth. Systematic investigations into the evaporation performances of these 3D evaporators revealed that the Z‐type evaporator stood out with an impressive evaporation rate of 4.42 kg m^−^^2^ h^−^^1^. Significantly, the Ag‐CCS based Z type evaporator demonstrated remarkable outdoor performance. It achieved a maximum daily water production of 25.1 kg m^−^^2^, accompanied by excellent automatic salt scaling cleaning capabilities, enabling it to maintain continuous operation for 20 days outdoors without performance degradation. The technique of using glue to construct evaporators with multiple 3D structures has been validated as a feasible, convenient, and cost‐effective strategy. This not only enriches the methodological toolkit for fabricating solar‐driven interfacial evaporators but also paves the way for their large‐scale development and practical applications.

## 2 Experimental Section

### 2.1 Materials and Chemicals

Waste corn shuck (CS) was carefully collected from a cornfield located in Yongxingzhuang Village, Qicun Town, Xinzhou City (Shanxi, China), then thoroughly washed, ground into a fine powder, sieved through an 80‐mesh sieve, and dried at 60°C for 24 h. NaOH was supplied by Tianjin Damao Chemical Reagent Factory. AgNO_3_ was supplied by Tianjin Fengchuan Chemical Reagent Technology Co., Ltd. TiO_2_ was supplied by Shanghai Macklin Biochemical Co., Ltd. Seawater was collected from the Yellow Sea near Qingdao, Shandong, China. All commercial reagents were used directly as received.

### 2.2 Preparation of CCS, Ag‐CCS, and Ti‐CCS

The collected CS powder was soaked in a prepared NaOH solution (0.2 mol L^−1^) for 24 hours, then dried in a air drying oven at 100°C for 24 h. Subsequently, it was placed in a vacuum box atmosphere furnace (KSX3‐4‐12, Hangzhou Zhuochi Instrument Co., LTD, China) and heated to 600°C at a rate of 5°C min^−1^ under N_2_ atmosphere for 2 h, and the treated sample was collected after cooling to room temperature. The obtained sample was named carbonized corn shuck (CCS). The prepared CCS was soaked in AgNO_3_ solution (0.2 mol L^−1^) and TiO_2_ solution (0.2 mol L^−1^) for 24 hours respectively. After drying by an air drying oven, the Ag‐CCS and Ti‐CCS were got by pyrolysis at 500°C for 1 h under N_2_ atmosphere using the same vacuum box atmosphere furnace.

### 2.3 Characterizations

The morphologies and microstructures of CCS, Ag‐CCS and Ti‐CCS were examined using a field emission scanning electron microscope (SEM, Zeiss Sigma 300, Germany) after gold sputter coating. The specific surface area and pore structure were tested by a Micromeritics ASAP 2460 apparatus (USA) with approximately 100 mg of samples. The FTIR spectra of the CCS based carbon materials were determined by a Fourier transform infrared spectrophotometer (FTIR, Bruker TENSOR II, Germany). The hydrophilic property of the samples was assessed via a contact angle meter (JY‐82C, Chengde Dingsheng Testing Machine & Equipment Co., Ltd., China). Furthermore, the concentrations of K^+^, Na^+^, Ca^2+^, Mg^2+^, and Li^+^ were determined using an inductively coupled plasma optical emission spectrometer (ICP‐OES, Agilent 5110, USA). Additionally, absorption spectra of both wet and dry samples were obtained by a UV‐visible‐NIR spectrometer over a wavelength range of 200–2500 nm (RG‐5000J, Tianjin Nuolei Xinda Technology Co., Ltd., China).

### 2.4 Fabrication Process of Different Shaped Evaporators

To achieve the goal of material conservation and controllable fabrication of evaporators with various geometric shapes, four typical geometric configurations (hemisphere, cone, flaky, Z‐type) were selected. To ensure the structural stability and durability of these configurations, PVC plastic or foam was adopted as the internal support skeleton. After wrapping the four support cores with absorbent cotton cloth, a water‐based adhesive was uniformly coated onto their surfaces, followed by closely attaching the as‐prepared photothermal conversion material onto the adhesive‐coated surfaces. After the carbon powder coating process was completed, the entire device was thoroughly dried to ensure it reached an optimal dry state prior to testing.

### 2.5 Solar Evaporation Experiment

All solar water steam generation performances were conducted under the illumination of a Xenon lamp (CME‐Sol1A300, Microenerg, Beijing, China), equipped with an AM 1.5 filter to accurately simulate solar irradiance. The solar intensity on the top surface of all samples was calibrated to 1000 W m^−2^ using a power meter (FZ‐A, Beijing Normal University, China). It should be noted that the average speed within each three minutes period was calculated as the evaporation rate, and the evaporation area used in the calculation was the projected area of each 3D evaporator. To evaluate the water evaporation performance of the solar absorber, the weight loss of the cylindrical container was monitored over time using a computer controlled electronic balance. Moreover, an infrared thermograph (UTi320E, UNI‐Trend Technology (China) Co., Ltd.) was used to track the temperature changes of the evaporator during the evaporation process, and a sealed transparent box was employed to collect condensed water outdoors for evaluating the actual performance of pure water and seawater evaporation.

### 2.6 Data Analysis

To evaluate differences in evaporation rate, energy efficiency, and long‐term stability among 2D and various 3D‐shaped evaporators, statistical analysis was performed using OriginPro (OriginLab Corporation, USA) and Microsoft Excel software (Microsoft, USA).

## 3 Results and Discussion

### 3.1 CS‐Based Photothermal Conversion Materials

**FIGURE 1:**
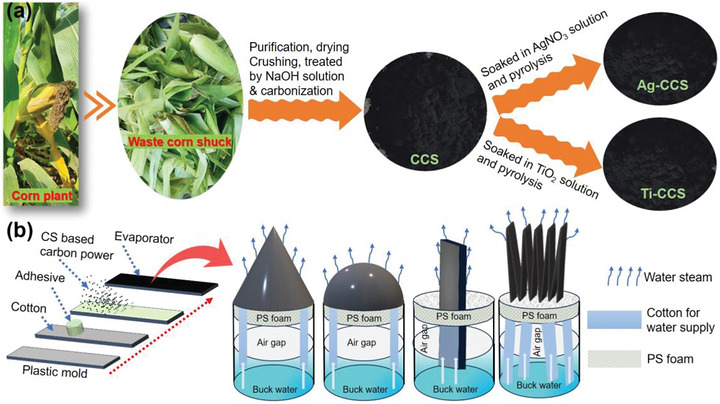
(a) Image illustration of preparation process of the CCS, Ag‐CCS and Ti‐CCS; (b) Schematic depicting the preparation procedure of various shaped 3D evaporators.

The fabrication process of the CS‐based photothermal conversion materials and the preparation process of the designed different shaped evaporators are exhibited in Figure 1. As shown in Figure 1a, the prepared CS powder was immersed in NaOH solution, and then carbonized in a vacuum furnace at 500 °C, resulting in the formation of CCS. The obtained CCS was soaked by AgNO_3_ and TiO_2_ solutions, respectively, and pyrolyzed at 500°C for 1 h to produce Ag‐CCS and Ti‐CCS. As exhibited in Figure 1b, four geometric configurations were selected for the fabrication of 3D versatile evaporators. PVC plastic or PS foam was used as the internal support to ensure thermal insulation, structural stability, and durability. After wrapping with cotton cloth, a water‐based adhesive and CS‐based carbon powder were uniformly coated on the surface of the cotton cloth.

**FIGURE 2:**
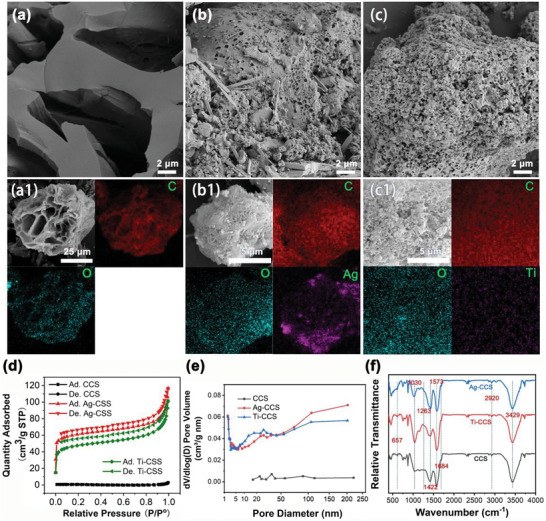
(a–c) SEM and EDS images of CS‐based carbon materials, where a, b, c represents CCS, Ag‐CCS and Ti‐CCS, a1, b1, and c1 represents EDS mapping of the CCS, Ag‐CCS and Ti‐CCS, respectively. (d) N_2_ adsorption–desorption curves of CCS, Ag‐CCS and Ti‐CCS. (e) Pore‐size distribution curves of CCS, Ag‐CCS and Ti‐CCS. (f) FTIR spectra of CCS, Ag‐CCS and Ti‐CCS.

The microstructural characteristics of the CCS, Ag‐CCS, and Ti‐CCS samples were investigated via SEM and BET, as depicted in Figure 2. Specifically, the CCS sample exhibits a densely packed, tightly stacked structure devoid of any discernible pores with a BET area of 2.9135 m^2^ g^−1^, as evidenced by the micrographs presented in Figure 2a and d. After undergoing further pyrolysis with AgNO_3_ or TiO_2_, a remarkable change in the microstructure is evident. Numerous pores emerge on the skeleton of the material, clearly demonstrated in the SEM images displayed in Figure 2b and c for Ag‐CCS and Ti‐CCS, respectively. The corresponding BET surface area increased to 233.3215 and 179.5394 m^2^ g^−1^ for sample CCS for Ag‐CCS and Ti‐CCS, respectively (compared to CCS). As exhibited in Figure 2e, the main pore size distribution of the CCS is from 16 to 220 nm; by comparison, the pore sizes of Ag‐CCS and Ti‐CCS decreased to approximately 1.9 nanometers, and the main distribution range was below 50 nm. This alteration in porosity suggests a significant impact of the pyrolysis process with the additives (AgNO_3_ and TiO_2_) on the microstructural features of the CCS samples. Figure 2a, 1, b1, c1 show the corresponding EDS mapping of the CCS, Ag‐CCS and Ti‐CCS. Based on the analysis of the EDS spectra, it is evident that the Ag element within the Ag‐CCS tends to partially agglomerate on the superficial layer of the carbonaceous material, as visually depicted in Figure 2b, 1. In contrast to the observed behavior in Ag‐CCS, Ti‐CCS exhibit a notably uniform distribution of Ti doping throughout the structure. As shown in Figure 2f, the FTIR spectra of CCS, Ag‐CCS, and Ti‐CCS are similar in their peak positions. The peaks at 3429 and 2920 cm^−1^ could be attributed to the stretching vibrations of the OH group and C─H bonds [39]. The peaks of 1684, 1573, and 1422 cm^−1^ are ascribed to the stretching vibrations of carbonyl C═C, O─C═O, and O─H groups [40]. The peaks of 1263, 1030, and 657 cm^−1^ are due to C─O, C═O and ─COOH [41]. Additionally, as summarized in Table 1, the elemental compositions on the surface of CS‐based carbon materials are quantified, revealing that the oxygen content in CCS, Ag‐CCS and Ti‐CCS is registered at 23.80, 20.02 and 19.18 wt%, respectively. Adequate oxygen content is the key to the excellent hydrophilic properties of the prepared materials. Furthermore, the Ag and Ti content for Ag‐CCS and Ti‐CCS are 12.43 and 0.20 wt%, respectively, further confirming the successful doping of silver and titanium in the carbon materials. It is noteworthy that the Ag‐CCS sample contains 1.19% of N element, potentially introduced by nitrate ions. These findings emphasize the significant impact of pyrolysis processes and additives on the microstructure and chemical properties of carbon materials.

**TABLE 1:** Surface element content of CCS, Ag‐CCS, and Ti‐CCS.

Sample	Elements/weight%				
	C	O	N	Ag	Ti
CCS	76.20	23.80	0	—	—
CCS‐Ag	66.36	20.02	1.19	12.43	—
CCS‐Ti	80.62	19.18	0	—	0.20

**FIGURE 3:**
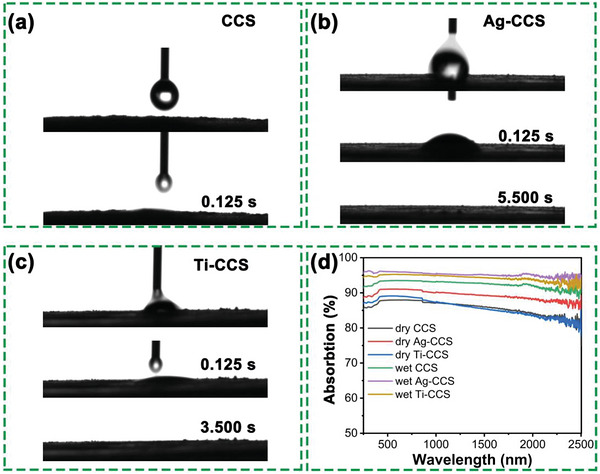
(a–c) Digital photo of water drop angle testing for samples CCS, Ag‐CCS, and Ti‐CCS, respectively; (d) UV–vis‐NIR absorption spectra of CS‐based carbon materials.

The hydrophilic characteristics exhibited by the resultant CS‐based materials are comprehensively depicted in Figure 3a–c. It is noteworthy that all the samples displayed prominent wettability, as manifested by the rapid disappearance of water droplets within 5.5 seconds. This observation underscores the inherent hydrophilic nature of these materials. In contrast, CCS exhibited exceptional superhydrophilic behavior, where water droplets were instantaneously absorbed within the imaging interval, highlighting its superior wettability. However, a slight decrement in wetting performance was observed for the silver‐loaded carbon‐coated chitosan (Ag‐CCS) and titanium‐loaded carbon‐coated chitosan (Ti‐CCS) samples. Specifically, water droplets on Ag‐CCS and Ti‐CCS required a short duration (5.5 seconds for Ag‐CCS and 3.5 seconds for Ti‐CCS) to be completely absorbed, indicating a moderately less efficient wetting mechanism compared to pristine CCS. The observed trends in hydrophilicity are in good agreement with the respective surface oxygen contents of these materials, as summarized in Table 1. This correlation suggests that an increase in surface oxygen content is positively associated with enhanced hydrophilic properties.

The exceptional solar light absorption capability of photothermal materials is a pivotal factor in achieving high speed evaporation and good energy conversion efficiency [34, 42]. To further investigate the light absorption behaviors of the CS‐based carbon materials, a UV–vis‐NIR spectrophotometer was employed to assess their solar light absorption capabilities under both dry and wet conditions. As illustrated in Figure 3d, under dry conditions, the CCS samples exhibited a modest light absorption of 85.69% across the broad UV–vis‐NIR spectral range, spanning from 250 to 2500 nm. Intriguingly, the incorporation of silver nanoparticles onto the CCS surface led to a substantial enhancement in light absorption, with an increase of 4.18%, elevating the overall absorbance to 89.27%. Conversely, the light absorption performance of Ti‐CCS remained virtually unaltered, with an absorbance value recorded at 85.73%. When the samples were subjected to wet conditions, a marked improvement in their light absorption capabilities was observed. For instance, the light absorption of wetted CCS, Ag‐CCS, and Ti‐CCS increased to 92.41%, 95.23%, and 94.26%, respectively. These results emphasize the significant impact for wetting condition of the light absorption properties for obtained materials, and highlight their potential application in solar‐driven interfacial water evaporation.

### 3.2 Photothermal Conversion Properties of the CCS Based Materials

**FIGURE 4:**
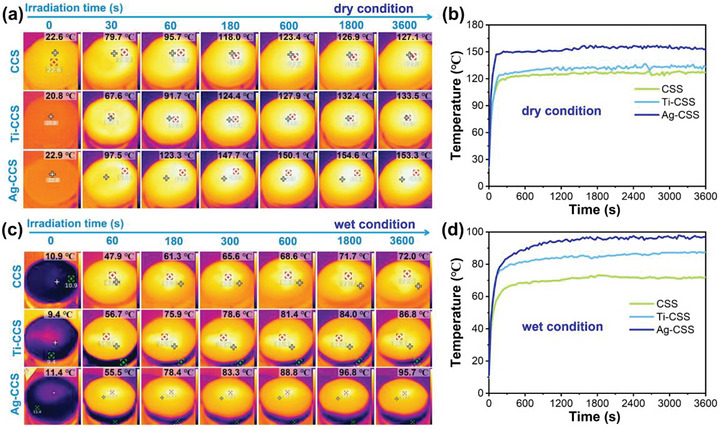
The surface temperature dependent illumination time for of CCS based evaporators under one sun (1000 W m^−2^). (a) Infrared thermal images of CSS based samples under dry condition. (b) Temperature rise curve of the CCS‐based evaporator under dry condition. (c) Infrared thermal images of CSS based samples under wet condition. (d) Temperature rise curve of the CCS‐based evaporator under wet condition.

To evaluate the photothermal conversion performance of the prepared CCS‐based materials, traditional 2D solar‐driven interfacial evaporators were fabricated, and their temperature changes on both dry and wet surfaces were tracked using an infrared thermal imager under one sun (1000 W m^−2^) simulated sunlight conditions. As shown in Figure 4a and b, the dry surface temperature of the dry Ag‐CCS undergoes a rapid and remarkable ascent. Specifically, within 30 seconds, its dry surface temperature rises from the ambient temperature of 22.9°C to 97.5°C, with the temperature increments relative to CCS and Ti‐CCS being 17.8°C and 29.9°C, respectively. Subsequently, over the next 30 seconds, the temperature of the dry Ag‐CCS further elevates to 123.3°C, demonstrating a higher value than those of CCS and Ti‐CCS by 27.6°C and 31.6°C, respectively. Surprisingly, the final temperature of the sample Ag‐CCS slowly increased to 153.3°C after one hour. Significantly, this high temperature was much higher than that of both CCS (127.1°C) and Ti‐CCS (133.5°C), which was in good agreement with the trend manifested in the UV–vis‐NIR spectra of these three materials. Investigating the photothermal conversion performance of wetted evaporators, particularly under operational conditions where they are wetted by water, is equally imperative. Our findings reveal that the photothermal conversion efficacy in the wet state mirrors that observed in the dry state. As shown in the Figure 4c and d, upon exposure to one‐sun irradiation, the wet surface of Ag‐CCS experiences a swift temperature surge, rising from approximately 11°C to 55.5°C within 60 seconds. After sustained illumination for 10 minutes, the wet surface temperature of Ag‐CCS escalates rapidly to 83.3°C, surpassing those of CCS and Ti‐CCS by 20.2°C and 7.4°C, respectively. In the subsequent 50 minutes, the wet surface temperature of Ag‐CCS gradually increases to 95.7°C, with the ultimate stable wet surface temperature of Ag‐CCS being 23.7°C and 8.9°C higher than those of CCS and Ti‐CCS, respectively. One phenomenon worthy of attention is that, under the same indoor conditions, the initial temperature of the wet samples is lower than that of the dry samples. This is mainly attributed to the endothermic effect caused by the evaporation of water on the surface of wet samples. The above results indicate that the porous structure on the surface of carbon materials as well as the presence of Ag and Ti (Figure 2) contribute to the capture and absorption of light, thereby significantly improving their photothermal conversion efficiency.

### 3.3 Solar Steam Generation of 2D and 3D Evaporators

**FIGURE 5:**
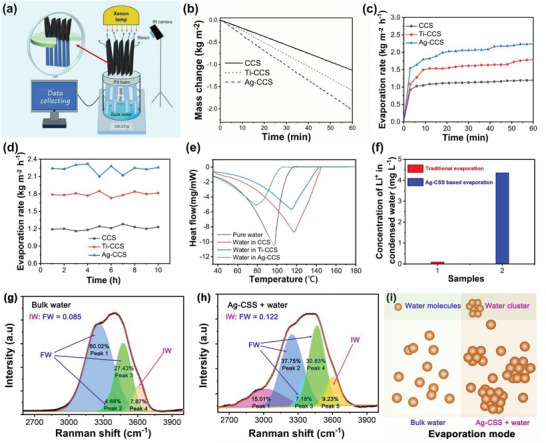
(a) Schematic illustration of the indoor solar driven water vapor generation test system; (b) Temporal dependence of water mass variation under one‐sun irradiation for CCS based evaporator; (c) Irradiation time‐dependent evaporation rate of CCS‐based evaporator under one‐sun; (d) The comparison of the evaporation rate of CCS‐based evaporator under one‐sun for long time; (e) DSC curves of the bulk water and absorbed water in CCS based evaporator; (f) The compare of Li^+^ concentration for treated water by Ag‐CCS and traditional evaporation; (g) Raman spectra of the bulk water; (h) Raman spectra of the interfacial water on Ag‐CCS; (i) Evaporation mode for bulk water and water on Ag‐CCS.

For systematically investigating the evaporation performance of the CS‐based carbon materials, a manually fabricated system was designed and utilized to evaluate the solar steam generation performance of resulting 2D and 3D evaporators, where the environment temperature and humidity were maintained at approximately 25°C and 40%, respectively. As illustrated in Figure 5a, the CS‐based carbon materials served as the photothermal converters, PS foam acted as a thermal insulation barrier, and non‐woven cotton fabric facilitated water transport. It should be noted that CS‐based carbon powder is integrated with cotton fabric through the use of an adhesive. Using cotton fabric as a carrier for CS based carbon powder can facilitate the construction of various shapes because of its controllable extensibility and flexibility, thereby overcoming the limitations imposed by the original form of the photothermal conversion materials.

The evaporation performance of water within the 2D evaporator system under investigation is comprehensively illustrated in Figure 5b and c. Within one hour, the evaporator with CCS underwent a mass reduction of 1.13 kilograms per square meter. Notably, when the evaporator was equipped with Ti‐CCS, the mass reduction escalated to 1.59 kilograms per square meter. Furthermore, the evaporator featuring Ag‐CCS exhibited an even more pronounced mass reduction, amounting to 2.06 kilograms per square meter. As shown in Figure 5c, the evaporation rate reaches 1.19, 1.79, 2.24 kg m^−2^ h^−1^ for CCS, Ti‐CCS and Ag‐CCS, respectively, after one hour of illumination under one sun. To evaluate the stability of the evaporation performance of CSS‐based evaporators, a continuous 10 hours evaporation experiment was conducted. As exhibited in Figure 5d, the evaporation rate of the CSS‐based evaporator is stable and does not show significant fluctuations. As previously reported in the literature [43], the theoretical maximum evaporation rate is 1.47 kg m^−2^ h^−1^. Apparent energy utilization refers to the calculation of energy efficiency by dividing the actual evaporation rate by the theoretical maximum evaporation rate, without considering the actual enthalpy change of water evaporation and energy losses. According to this method, the apparent energy utilization rates are 80.9%, 121.8%, and 152.4% corresponding to CCS, Ti‐CCS and Ag‐CCS, respectively.

The above results seem incorrect, as the evaporation rate far exceeds the theoretical value. We overlooked an issue here: which is that the enthalpy of evaporation of water may be altered when adsorbed by photothermal conversion materials [7, 43]. For this reason, the enthalpy of evaporation of adsorbed water corresponding to CCS, Ti‐CCS and Ag‐CCS was tested by a differential scanning calorimeter (DSC). As illustrated in Figure 5e, the vaporization enthalpy of bulk water was determined to be 2471.52 J g^−1^, which is very close to the reported value [7]. The vaporization enthalpy of interfacial water in CCS was approximately 2397.15 J g^−1^, nearly equivalent to that of bulk water. However, a significant reduction in vaporization enthalpy was observed for water in Ti‐CCS and Ag‐CCS, with values decreasing to 1683.94 and 1286.13 J g^−^^1^, respectively. Based on these DSC results, the corresponding energy efficiencies for water evaporation in Ti‐CCS and Ag‐CCS were calculated to be approximately 83.7% and 80%, respectively. Apparently, the observed high evaporation rate is predominantly attributable to the low enthalpy of adsorbed water within Ag‐CCS.

To further validate this mechanism of lower enthalpy of evaporation for Ag‐CCS, LiCl solutions with a concentration of 80 g L^−1^ were evaporated using Ag‐CCS, following a methodology similar to that employed in previous studies [44]. Figure 5f reveals that the condensed water evaporated by Ag‐CCS contained 4.360 mg L^−1^ of Li^+^, whereas the black sample contained only 0.102 mg L^−1^ of Li^+^. This result conclusively confirms that water evaporation by Ag‐CCS occurs predominantly in the form of small water clusters. In the Raman spectra of water, the intensity of the signal corresponding to free water (FW) within the 3200–3500 cm^−1^ spectral range serves as an indicator of the proportion of unconstrained water molecules. Conversely, the intensity of the signal associated with intermediate water (IW) in the 3500–3700 cm^−1^ spectral region reflects the proportion of confined water molecules [43, 45, 46]. As illustrated in Figure 5g and h, the IW/FW ratio for bulk water was measured to be 0.085, while for water on Ag‐CCS, this ratio increased to 0.122. The increase in the proportion of IW can be attributed to the presence of a large number of hydrophilic nanopores on the surface of Ag‐CCS. IW facilitates the evaporation of water in the form of clusters. As shown in Figure 5i, water evaporation in the form of small water clusters requires a lower enthalpy change and less energy. Evaporation in the form of clusters can mitigate the energy required to disrupt the interaction forces between water molecules inside the clusters [44].

**FIGURE 6:**
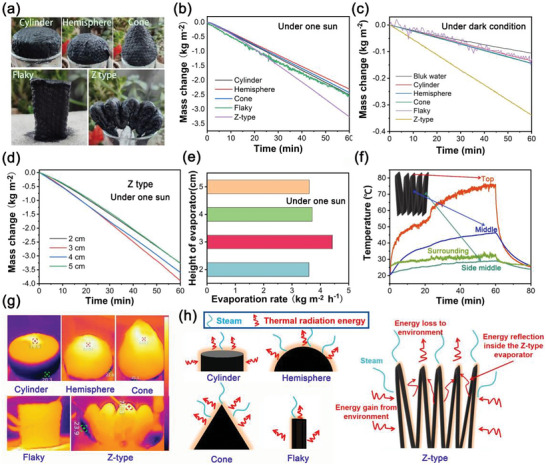
(a) Optical images of Ag‐CCS based evaporator with the shape of cylinder, hemisphere, cone, flaky, Z‐type; (b) Time dependence of water mass variation under one‐sun irradiation for different shape evaporators; (c) Time dependence of water mass variation under dark condition for different shape evaporators; (d) Time dependence of water mass variation under one‐sun irradiation for different height Z‐type evaporators; (e) Average evaporation rate of the different height evaporator for Z‐type in final 5 min; (f) Time dependence of temperature under one‐sun irradiation for Z‐type evaporators; (g) Infrared thermal images for different shape evaporators; (h) Energy flow schematic diagrams for different shape evaporators.

Considering that 3D evaporators have numerous advantages, including an augmented evaporation surface area, improved solar energy capture, and the capacity to extract energy from the surrounding environment [47, 48, 49]. A series of 3D evaporators with different shapes were prepared to further enhance the evaporation rate. As exhibited in Figure 6a, cylinder, hemisphere, cone, flaky, Z‐type evaporators were prepared for exploring the relationship between evaporation performance and 3D shapes. Figure 6b shows the variation of mass over time for different shape Ag‐CCS based evaporators with a length of 2 centimeters. Within one hour, the mass reductions of the evaporators were observed to be 2.50, 2.30, 2.42, 2.55, and 3.26 kg m^−2^ h^−1^ for cylinder, hemisphere, cone, flaky, Z‐type evaporators, respectively. The corresponding energy efficiencies of cylinder, hemisphere, cone, flaky, Z‐type evaporators were calculated to be approximately 97.1%, 89.3%, 93.9%, 99.0%, and 126.6%, respectively. It is evident that the performance of the fabricated 3D evaporator outperforms that of the 2D evaporator.

To further investigate the impact of height on the evaporation performance of Z‐type evaporators, evaporators with heights of 2, 3, 4, and 5 cm were prepared herein. As exhibited in Figure 6c, over one hour, the weight reductions of evaporators with heights of 1, 2, 3, and 4 cm were 3.26, 3.90, 3.59, and 3.27 kg m^−2^, respectively. To better compare the performance of evaporators at various heights, the evaporation rates were calculated based on the final five minutes. As shown in Figure 6d, the evaporation rates were reached up to 3.59, 4.42, 3.71, and 3.61 kg m^−2^ h^−1^ for Ag‐CCS based evaporators with heights of 1, 2, 3, and 4 cm, respectively. It is evident that the evaporation performance initially increases and then decreases with increasing height, with the Z‐type evaporator at a height of 2 cm exhibiting the optimal evaporation performance. The likely cause of this phenomenon is the synergistic effect between the water transport speed (determined by height) and the water evaporation rate. At relatively low heights, the transport of water is excessive compared to the evaporation capacity. As a result, not all the transported water can evaporate, leading to water accumulation. Conversely, at overly high heights, the water transport speed cannot meet the demands of evaporation. This imbalance between water supply and evaporation demand, caused by height‐related changes in water transport, contributes to the observed phenomenon.

To elucidate the underlying mechanisms contributing to the high evaporation rate of the Z‐ ttype evaporator, a systematic investigation was conducted on ambient energy utilization capacity (beyond solar radiation) and thermal management mechanisms. As shown in Figure 6c, on dark condition, the mass reduction is 0.11 kg m^−2^ for bulk water for 1 h. In contrast, the Z‐type evaporator presented the most significant mass reduction, with its mass change reaching 0.34 kg m^−2^ at 60 min, far exceeding that of bulk water. The cylinder, hemisphere, cone, and flaky evaporators also outperformed bulk water. The evaporation performance in dark environments can indirectly reflect the energy absorption performance from surrounding environment of evaporators with different shapes from the environment. For further evidence, thermal infrared imaging of evaporators with different shapes were measured under one sun. As shown in Figure 6g, the evaporators of hemisphere, cone, and flaky shapes exhibit surface temperatures distinctly higher than the ambient temperature. In contrast, the outer side temperature of cylindrical and Z‐shaped evaporators is lower than the ambient temperature, indicating that these two configurations can absorb energy. As depicted in Figure 6f, the Z‐type evaporator exhibits a top to centered thermal hierarchy: the top has the highest temperature, the internal side middle is hotter than the surrounding, and the outer side middle is the coolest. As shown in Figure 6h, energy flow diagrams characterizing evaporators with distinct geometric configurations are systematically constructed. The hemisphere, cone and flaky evaporators mainly lose heat through direct thermal radiation to the environment. The cylinder evaporator can harvest a certain amount of energy from the environment. In contrast, the Z‐type evaporator not only effectively gains energy from the environment but also utilizes internal energy reflection to reduce energy loss to the surrounding environment. This unique structural design enables the Z‐type evaporator to achieve better thermal localization and utilization, resulting in superior thermal management performance compared to the cylinder, hemisphere, cone, and flaky evaporators. Overall, the Z‐type structure shows significant advantages in thermal management, providing higher evaporation rate.

**FIGURE 7:**
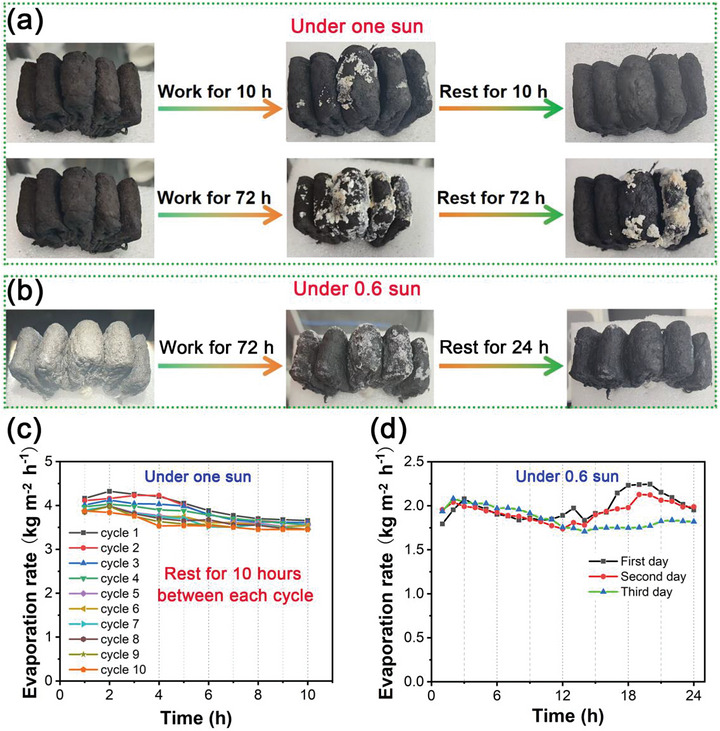
(a) Photo images of salt deposition and automatic cleaning performance under one sun; (b) Photo images of salt deposition and automatic cleaning performance under 0.6 sun; (c) Time dependence of evaporation rate under one‐sun irradiation for Z‐type evaporator, between each cycle rest for 10 h; (d) Time dependence of evaporation rate under one‐sun irradiation for Z‐type evaporator, where the Z‐type evaporator no rest during the test period.

To evaluate the salt resistance, stability, and durability of the Z‐type evaporator, we conducted investigations under illumination intensities of 1 and 0.6 sun while treating the system with a 7 wt% NaCl solution. As shown in Figure 7a, under 1 sun illumination, after 10 h of work followed by 10 h of rest, the evaporator could regain a relatively clean surface, and the evaporation rate during 10 cycles (with 10 h rest between each cycle) remained stable at approximately 3.5–4.0 kg m^−2^ h^−1^ (Figure 7c), indicating good cyclic stability. However, when the evaporation duration was extended to 72 h under 1 sun, significant salt accumulation occurred on the evaporator surface. Even after 72 h of rest, complete salt removal was not achieved. Under 0.6 sun (Figure 7b), after 72 h of evaporation and 24 h of rest, the evaporator also showed salt accumulation, but the surface could largely recover. The evaporation rate under 0.6 sun exhibited slight fluctuations over three days of cyclic testing (Figure 7d) but generally maintained a level of 1.5–2.0 kg m^−2^ h^−1^, suggesting acceptable long term cyclic performance under 0.6 sun solar intensity. Overall, the evaporator exhibits good cycling stability, and according to the simulated natural conditions of 12 hours of light exposure and 12 hours of rest, it can achieve the self‐cleaning performance of salt.

### 3.4 Outdoor Performance and Durability

**FIGURE 8:**
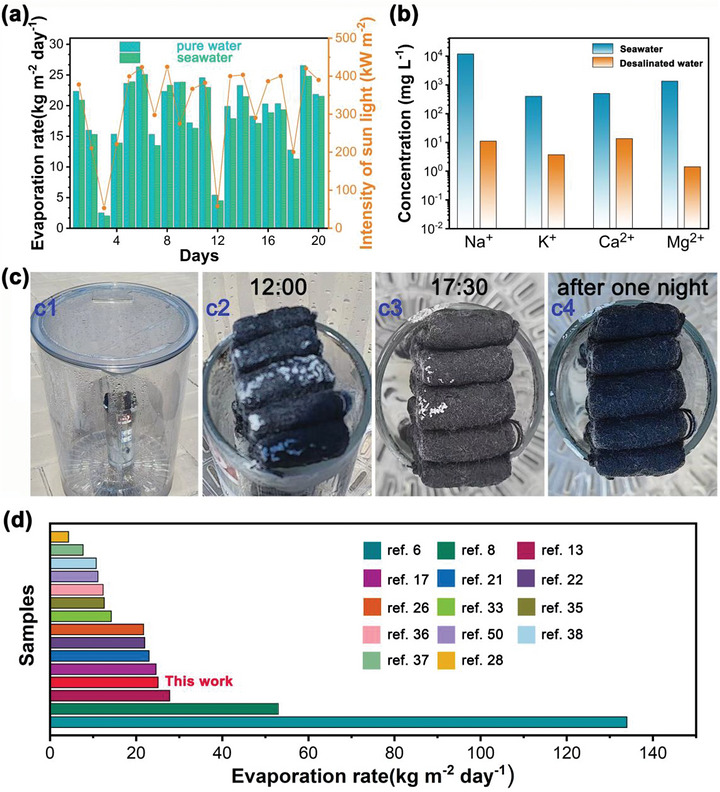
(a) Outdoor water steam production performance of the Ag‐CCS based Z type evaporator over a 20‐day period; (b) The concentration of Na^+^, K^+^, Ca^2+^, and Mg^2+^ irons in the seawater from the Yellow Sea, before and after treatment using an Ag‐CCS‐based Z‐type evaporator in an outdoor setting; (c) Image depicting the outdoor solar seawater desalination test system and salt deposition and automatic cleaning process; (d) The comparison of the evaporation rate of our Z‐type evaporator with other reported solar‐driven evaporators.

The working stability and practical performance serve as crucial indicators for evaluating the efficacy of solar interfacial evaporators. In light of this significance, an elaborate outdoor test setup was meticulously designed, as vividly depicted in Figure 8c, 1. These outdoor tests were carried out at Xinzhou Teachers University, located in Xinfu District, Xinzhou City, Shanxi Province. Over a continuous period of 20 days, a comprehensive tracking of the water evaporation performance of the Ag‐CCS based Z‐type evaporator was executed, with both deionized water and Yellow Sea seawater serving as the test media. As illustrated in Figure 8a, the Ag‐CCS based Z‐type evaporator demonstrated remarkable stability throughout a continuous 20 days period. For deionized water, the average daily evaporation amount reached 18.9 kg m^−^^2^, while for Yellow Sea seawater, it was 17.9 kg m^−^^2^. Significantly, these evaporation yields are sufficient to meet the drinking water requirements of approximately 8–10 adults, highlighting the practical viability of this evaporator in water provisioning scenarios. It is important to note that the daily water production performance of the Ag‐CCS based Z‐type evaporator is closely intertwined with sunlight intensity. Generally, as the light intensity increases, the water production of the fabricated evaporator also experiences a corresponding enhancement. This relationship is a key factor in understanding the evaporator's efficiency under real world sunlight irradiation conditions. Specifically, the daily evaporation rate of seawater using this evaporator exhibits a notable fluctuation, ranging from 2.02 to 25.1 kg m^−^^2^. As shown in Figure 8d, the Z‐type evaporator exhibits excellent outdoor evaporation performance compared with the highest values reported in the literature [7, 8, 13, 17, 21, 22, 26, 28, 33, 35, 36, 37, 38, 50]. As depicted in Figure 8b, after treatment of the Yellow Sea seawater by the Ag‐CCS based Z‐type evaporator, the ion concentrations of Na^+^, K^+^, Ca^2+^, and Mg^2+^ decreased significantly, from the initial concentration of 12008, 404, 505, 1360 to 11.1, 3.8, 13.7, 1.4 mg L^−1^, respectively. This remarkable decrease in ion concentrations highlights the high efficiency of the Ag‐CCS based Z‐type evaporator in purifying seawater. According to the World Health Organization (WHO) standards [28, 51], the quality of the treated water meets the criteria for drinking water. Additionally, Ag‐CCS based Z‐type evaporator exhibits a highly valuable automatic salt cleaning capability. This property is of particular significance in seawater desalination applications. As shown in Figure 8c, during the process of treating seawater outdoors, large amounts of crystalline salt emerge on the surface of the Z‐type evaporator after working for 4 h (at am 12:00, Figure 8c, 2). Most of the salt crystals disappear at 17:30 (as shown in Figure 8c, 3), and after one night of rest, the salt crystals on the surface of the evaporator completely disappear automatically (as shown in Figure 8c, 4). This phenomenon can be adequately elucidated based on the framework of chemical potential theory. According to this theory, the surface chemical potential of salt exceeds that of bulk water. The driving force (chemical potential) arising between the evaporator and the bulk salt water facilitates the migration of salt back into the bulk water [52]. As shown in the Figure S1 and Table S1 in the Supporting Information, Ag nanoparticles remain stably distributed on the evaporator surface without obvious agglomeration, migration. Overall, the prepared Z‐type evaporator demonstrates remarkable efficiency in purifying seawater with ability of automatic cleaning sedimentary salt, simplifying the maintenance and operation of desalination systems.

## 4 Conclusions

In summary, we have developed a versatile method to fabricate 3D solar‐driven evaporators with diverse shapes (hemisphere, cone, flaky, Z‐type) by bonding corn shuck‐derived carbon powder onto cotton cloth with binders. The Z‐type evaporator exhibits an optimal evaporation rate of 4.42 kg·m^−^^2^·h^−^^1^ under 1‐sun illumination, outperforming 2D counterparts and other 3D shapes. This superiority is attributed to the synergistic effects of Ag‐CCS's porous structure, high photothermal conversion efficiency, and reduced evaporation enthalpy for interfacial water. Moreover, the Z‐type structure enables efficient ambient energy absorption and internal energy reflection, creating a thermal hierarchy that enhances heat localization. This design reduces energy loss and improves thermal efficiency compared to traditional shapes. Outdoor experiments demonstrate the Z‐type evaporator's daily water production of 25.1 kg·m^−^^2^ for seawater desalination with auto cleaning capability. The binder‐based fabrication strategy overcomes the shape constraints of traditional carbon‐based evaporators, enabling arbitrary 3D structures with low cost and scalability. This approach broadens the horizon for developing efficient solar‐driven systems for freshwater production.

## Author Contributions

**Xidong Suo**: Investigation, data curation, visualization, writing – original manuscript, writing – review & editing. **Yufan Yan**, **Jiayu Mu**, **Kaiyan Hao**, **Xintong Yu**: Indoor data investigation and outdoor data investigation. **Hongtao Qiao**: Investigation, data curation. **Jie Yang**: Methodology, conceptualization, software, validation, data curation, writing – original manuscript, writing – review & editing, funding acquisition, supervision.

## Funding

The authors would like to thank the funding support by: Science & technology plan and project in XinZhou (20230508), Fundamental Research Program of Shanxi Province (20210302124332), Shanxi Scholarship Council of China (2023‐165), Technology Plan Project in Xinzhou City of China (20230703).

## Ethical Approval

No animals were killed. There is no ethical approval needed in this work.

## Conflicts of Interest

The authors declare no conflict of interest.

## Supporting information




**Supporting File**: gch270113‐sup‐0001‐SuppMat.docx.

## Data Availability

The data that support the findings of this study are available on request from the corresponding author. The data are not publicly available due to privacy or ethical restrictions.
