# Supplementary material for: Shape‐Customizable 3D Corn Husk‐Derived Carbon Evaporator for High‐Performance Solar Desalination
Source: Glob Chall. 2026 May 7;10(5):e00002. doi: 10.1002/gch2.202600002 (PMC13150476; doi:10.1002/gch2.202600002)

Supporting Information

**Shape-customizable 3D corn husk-derived carbon evaporator for high-performance solar desalination**

Xidong Suo*, Yufan Yan, Jiayu Mu, Kaiyan Hao, Xintong Yu, Hongtao Qiao*, Jie Yang*

Department of Chemistry, Xinzhou Normal University, No. 1 Dun Qi Street, Xinzhou, Shan Xi, 034000, China

**Corresponding Author ：** Xidong Suo, Jie Yang, Hongtao Qiao


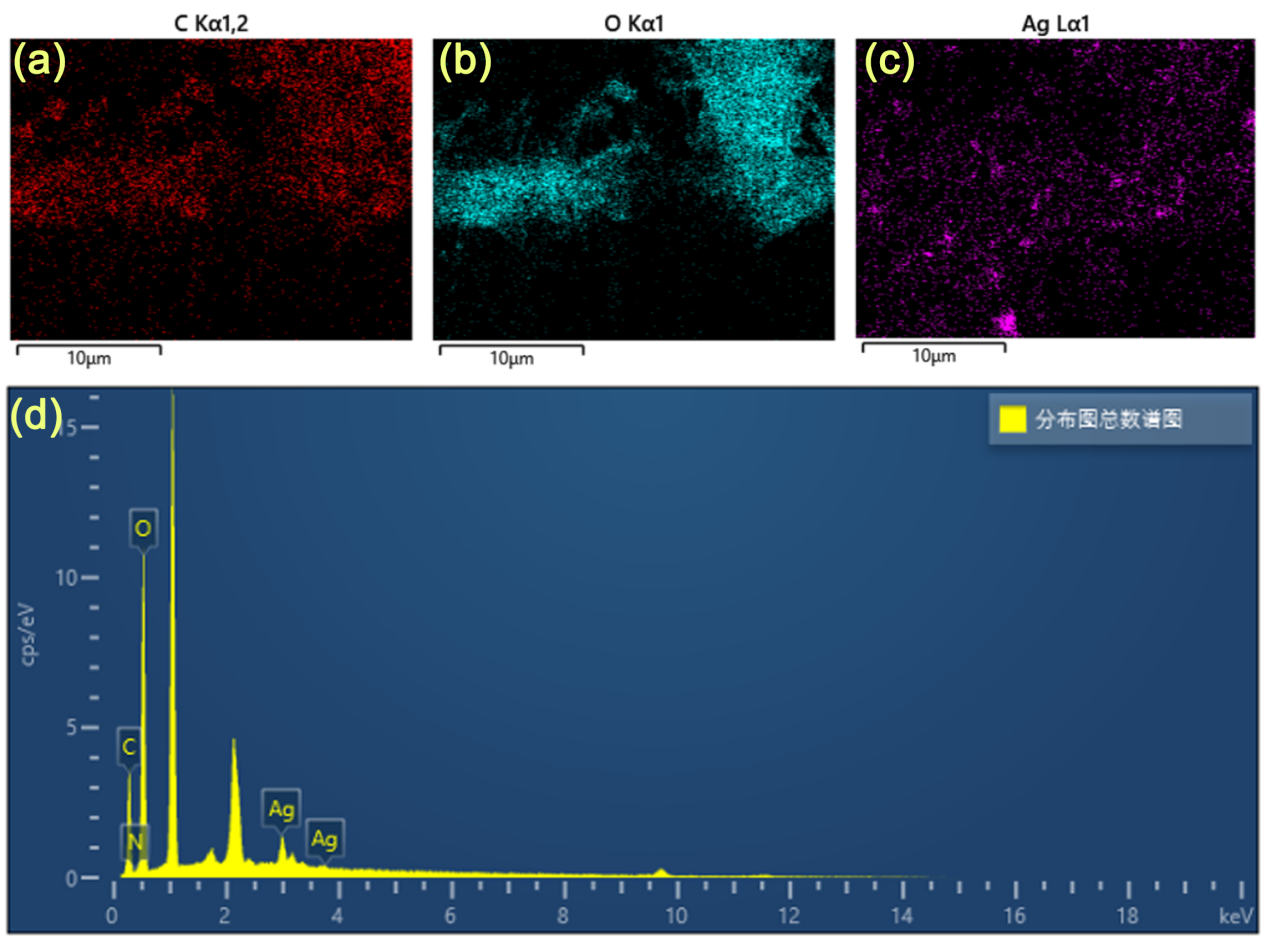
**Figure S1**. (a-d) EDS images of Ag-CCS after working 10 days.

**Table S1**. Surface element content of Ag-CCS after working 10 days.


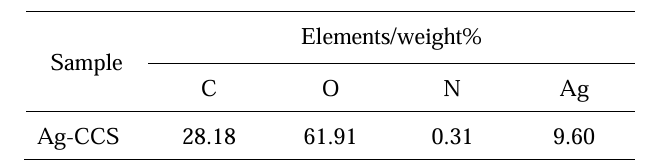

Supplement: Supplementary file 1 — Supporting File: gch270113‐sup‐0001‐SuppMat.docx. [file GCH2-10-e00002-s001.docx]
